# 

**Published:** 1830-04

**Authors:** 


### MONTHLY LIST OF MEDICAL BOOKS.

[*Medical Works cannot be entered on this List except a copy be sent for the purpose; the titles of Books having frequently been transmitted to us, as published, which have not appeared for weeks, or even months, after.*]

Medical Botany; or, Illustrations and Descriptions of the Medicinal Plants of the London, Edinburgh, and Dublin Pharmacopœias, with those lately introduced into Medical Practice; including a Popular and Scientific Description of Poisonous Plants, particularly those that are indigenous to Great Britain and Ireland, with Figures coloured from Nature. The whole forming a complete System of Vegetable Toxicology and Materia Medica. By JOHN STEPHENSON, M.D. F.L.S. &c. and JAMES M. CHURCHILL, F.L.S. &c. Tilt, Fleet street.—Monthly Numbers for December 1829, January, February, and March 1830.

We have but to repeat the opinion we have so frequently expressed of this elegant and useful work. The plates are excellent, and the descriptions of the various plants concise and practical.

Sketches of the Medical Topography of the Mediterranean; comprising an Account of Gibraltar, the Ionian Islands, and Malta: to which is prefixed, a Sketch of a Plan for Memoirs on Medical Topography. By JOHN HENNEN, M.D. F.R.S.E., Inspector of Military Hospitals, author of the "Principles of Military Surgery," &c. Edited by his Son, J. HENNEN, M.D., Mem. Roy. Med. Soc. Edin. &c.—8vo. pp. 666. Underwood, London, 1830.

Every officer, whether military or medical, who is stationed at either of the places mentioned in the titlepage, ought to be in possession of this most interesting and instructive work. These "Sketches" were originally drawn up in the form of Reports, and transmitted from time to time to the Director General of the Army Medical Department, for his information. The plan adopted by Dr. Hennen for describing the medical topography of the Mediterranean will serve as an excellent model for others who are prosecuting similar inquiries in other parts of the world. This elaborate work affords an additional proof of the indefatigable zeal, talent, and philanthropy of the late Dr. Hennen. To his son, the editor, the gratitude and approbation of the profession and the public in general are due, for the labour he has bestowed, and the ability he has shown in arranging and selecting the important materials left him by his much-lamented parent.

Observations on the existing Distinctions between Physic and Surgery; with Remarks on the general State of the Medical Profession. By KLEIN GRANT, M.D. &c.—8vo. pp. 24. Wilson, London 1830.

The absurdity of the existing distinctions between physic and surgery is ably pointed out by Dr. Grant. He contends that both branches are so intimately interwoven, that he who does not understand both, cannot possibly understand either. As a proof that no aristocratic pride belongs to Dr. Grant, we may quote the following just and liberal sentiment: "The heraldry of science admits no gradations of rank, but those which arise from intellectual superiority."

Hints for the Suppression and Extinction of Fires in Dwelling Houses, &c. and for the Preservation of Property in general from the Ravages of this destructive Element. Addressed to the Public in general, and to Fire Assurance Associations in particular. By ROBERT VENABLES, M.B.—pp. 16. Longman.

Although it is our business to check the flames of disease rather than of "fires," we may just stop to notice the proposal of Mr. Venables. He recommends that azotic or carbonic acid gas, either of which will arrest the process of combustion, should be collected in gasometers, and transferred in balloons of oiled silk or leather to the place on fire. "Once forced into an apartment or building, it would not flow off or evaporate like water, but would envelope the whole of the burning material with a non-supporting medium, and thus speedily extinguish the fire." It is worthy of observation, that these gases would not injure any species of property; while water, although it arrests the flames, destroys most of the property. One great, and indeed fatal, objection to the proposed plan, appears to have escaped Mr. Venables' notice: in most houses or buildings on fire, there is a ready admission for a large volume of atmospheric air, which would dilute and destroy the anti-combustible properties of either of the gases he recommends.

A Pocket Compendium of Anatomy, containing a correct and accurate Description of the Human Body. Second Edition. By E. W. TUSON, F.L.S. &c.—Wilson, London, 1830.

A concise and well-arranged book of reference for the anatomical student.

A Dissertation on the Influence of Heat and Humidity; with Practical Observations on the Inhalation of Iodine and various Vapours in Consumption, Catarrh, Croup, Asthma, and other Diseases. By JAMES MURRAY, M.D. &c. —8vo. pp. 305. Longman, London, 1829.

On the Nature and Treatment of the most frequent Diseases of Children; with Observations on the Management of early Infancy, Practical Remarks on the Exhibition of Opium, and on general and local Bleeding. By MILES MARLEY, F.L.S., Member of the Royal College of Surgeons.—8vo. pp. 312. Burgess and Hill, London, 1830.

Mr. Marley has given very slight sketches of most of the diseases incidental to childhood. The general contents of the book have been so frequently repeated by various writers, that their accuracy cannot be doubted. The manner in which the work is got up is creditable to the publisher.

A Treatise on Poisons, in relation to Medical Jurisprudence, Physiology, and the Practice of Physic. By ROBERT CHRISTISON, M.D., Professor of Medical Jurisprudence and Police in the University of Edinburgh, &c.—8vo. pp. 698. Black, Edinburgh; Longman, London, 1829.

We have already endeavoured to convey to our readers a general knowledge of this very important work in our Review. We shall from time to time enrich our Collectanea department with many interesting extracts from it.

Observations on the Disorders of Females connected with Uterine Irritation. By THOMAS ADDISON, M.D., Assistant Physician and Lecturer on the Theory and Practice of Physic at Guy's Hospital.—8vo. pp. 96. Highley, London, 1830.

A Letter to Sir Henry Halford, Bart., K.C.H., President of the Royal College of Physicians, &c., touching some Points of the Evidence, and Observations of Counsel, on a Commission of Lunacy on Mr. Edward Davies. By G. MAN BURROWS, M.D.—8vo. pp. 38. Underwood, London, 1830.

A Vade-Mecum of Morbid Anatomy, Medical and Chirurgical; with Pathological Observations and Symptoms. Illustrated by upwards of 250 Drawings.—8vo. pp. 51. Burgess and Hill, London, 1830.

This work must prove of great utility to practitioners and medical students, in whatever branch of the profession they may be engaged. The price of it is very moderate, considering the number and elegance of the numerous plates it contains. The arrangement is extremely convenient, either for occasional reference or regular perusal. The work contains, observations on, with illustrations of, the changes of structure found in the brain, thoracic, abdominal, and pelvic viscera, and of the organs of generation in both sexes. It likewise gives the pathological symptoms by which we judge of disease during life, and a true description of the morbid changes that are exhibited after death.

A Treatise on Hysteria. By George Tate, Member of the Royal College of Surgeons in London.—8vo. pp. 134. Highley, 1830.

### NOTICE TO CORRESPONDENTS.

*The Editor will reply to the several queries of his Birmingham friend without delay.*

*The project of* Dr. G. *looks well on paper, but it never can succeed in practice.*

*Communications have been received from* Mr. Wallaw, Dr. *and* Mr. Griffin.

